# Technological quality and fungal community of Kombucha fermented with hemp leaves and milky mushroom flour (*Calocybe indica*)

**DOI:** 10.7717/peerj.18116

**Published:** 2024-09-26

**Authors:** Priyada Sittisart, Thitikorn Mahidsanan, Vasin Yuvanatemiya, Pattama Srinamngoen

**Affiliations:** 1 Department of Agricultural Technology, Faculty of Science and Arts, Burapha University, Chanthaburi Campus, Chanthaburi, Thailand; 2 Department of Food Science and Technology, Faculty of Agricultural Innovation and Technology, Rajamangala University of Technology Isan, Nakhon Ratchasima, Thailand; 3 Faculty of Marine Technology, Burapha University, Chanthaburi Campus, Chanthaburi, Thailand

**Keywords:** Microbial consortium, Kombucha, Fermented beverage, Hemp leaves, Milky mushroom, Phytonutrients

## Abstract

Kombucha is traditionally a non-alcoholic beverage whose production is dependent on culture and the various ingredients used as substrates for fermentation. The goal of our study was to apply hemp leaf and milky mushroom (*Calocybe indica*) flour as functional ingredients to enhance phytonutrient quality, along with using a microbial consortium highly symbiotic with these ingredients. The study determined the content of phytonutrients (phenolic and flavonoids content), antioxidant activity through percentage inhibition of DPPH radical scavenging activity (%), and microbial communities changes during fermentation. The microbial changes were evaluated by cell viable count (total bacteria, Lactic Acid Bacteria, and Yeast & Mold) and ITS in prepared kombucha (using red tea leaves, pandan leaves, and sucrose) supplemented with functional ingredients: T1 (hemp leaves (control)) and T2 (hemp leaves with milky mushroom flour). The results indicated that microbial consortium changed during fermentation. In the first 7 days, the levels of yeast and mold increased to 6.17 and 6.18 log CFU/mL, respectively. By day 21, the levels of both T1 and T2 continued to rise, reaching 7.78 and 7.82 log CFU/mL, respectively. The viable count of lactic acid bacteria in T1 and T2 gradually increased to 6.79 and 6.70 log CFU/mL, respectively, by day 14. These changes resulted in a marked decrease in pH value, reaching 3.63 and 3.23 in T1 and T2, respectively, by the end of the process (21 days). The total bacterial viable count decreased with an increase in the fermentation time. During fermentation, unique genera of tea fungus observed in T1 and T2 were 64% and 19%, respectively. At the beginning (0 days), the top five genera found in T1 were: *g__Setophoma* (25.91%), *g__Macrocybe* (14.88%), *g__Cladosporium* (7.81%), *g__Phaeosphaeria* (7.12%), *g__Malassezia* (6.63%), while the top five genera in T2 were *g__Macrocybe* (94.55%), *g__Setophoma* (1.87%), *g__Cladosporium* (0.77%), *g__Phaeosphaeria* (0.40%), *g__Cordyceps* (0.38%). However, on day 21 (end of the process), it was found that *g__Dekkera* had the highest relative abundance in both T1 and T2. In addition, the supplementation of the two ingredients affected the total phenolic and total flavonoid content of the treatments. At the end of the process, T2 showed values of 155.91 mg GAE/mL for total phenolics and 1.01 mg CE/mL for total flavonoids, compared to T1, which had 129.52 mg GAE/mL and 0.69 mg CE/mL, respectively. Additionally, the DPPH inhibition was higher in T1 (91.95%) compared to T2 (91.03%). The findings suggest that kombucha fermented with these innovative ingredients exhibited enhanced phytonutrients, and served as substrate for LAB and tea fungus fermentation, while limiting the growth of fungal genera and diversity of microbial consortium.

## Introduction

Excessive alcohol consumption poses a major threat to global public health. Thereby, the World Health Organization (WHO) highlights that beyond health conditions like alcoholism, excessive drinking contributes to severe social consequences (Yoshimoto et al., 2023). Nowadays, non-alcoholic beverages, which offer flavors similar to alcoholic drinks but without the alcohol, could potentially benefit public health. In many countries, known non-alcoholic beverages like fermented beverages are a testament to the ingenuity of traditional food preservation. Throughout history, fermentation has extended shelf-life while simultaneously creating new flavors, textures, aromas, and nutritional value (Baschali et al., 2017). Kombucha is a traditional non-alcoholic fermented beverage (Batista et al., 2022). It typically features a sparkling, sweetened tea base, prepared through the fermentation of sugared tea infusion at room temperature over several days. The process uses a consortium of yeast, lactic acid bacteria (LAB), and acetic acid bacteria (AAB) that are found in cellulose pellicle, also known as a tea fungus (Matei et al., 2018). During the fermentation process of kombucha tea, a gelatinous film composed of cellulose forms at the interface between the air and the liquid. This film is known as a Symbiotic Culture of Bacteria and Yeast (SCOBY), which is a key product derived from the kombucha fermentation process (Al-Mohammadi et al., 2021). The microbial composition within a SCOBY can vary, but viable AAB and yeast are predominant. Typically, species of yeast isolates found in SCOBY belong to *Schizosaccharomyces pombe*, *Saccharomyces cerevisiae*, *Dekkera brucellosis*, *Pichia kudriavzevii*, *Brettanomyces animals*, *Starmerella vitis*, and *Hanseniaspora valbyensis*. The dominant AAB species are identified as *Acetobacter musti* and *Gluconobacter potus*, including *Lactobacilli/Lactococci* group, an important LAB, which is known for its probiotic potential health benefits (da Anunciação et al., 2024; Wang et al., 2022; Bogdan et al., 2018). Within this symbiotic culture, or tea fungus, various metabolic processes occur simultaneously across a gradient of oxygen levels. Among these processes are anaerobic ethanol fermentation by yeast, organic acid fermentation, and aerobic ethanol oxidation to acetate, which is facilitated by bacteria. Additionally, the microbial population includes acetobacter bacteria, which can polymerize glucose residues to form a solid-phase cellulosic pellicle mat that provides structural support for the microbial community. This production process takes place around temperatures between 22 °C to 25 °C for a period of 7 to 14 days (Villarreal-Soto et al., 2018; Jakubczyk et al., 2020). The symbiotic culture is prepared and available for acquisition or utilization in fermentation. Typically, these cultures or SCOBY, are utilized as by-products for commercial purposes. Currently, both small-scale beverage producers and households commonly utilize kombucha SCOBY to produce fermented tea. As we know, kombucha contains beneficial compounds derived from the raw materials and the oxidation of polyphenols during fermentation. Therefore, alternative raw materials for kombucha production, including fruits, herbs, milk, spices, sugar, and fungus, are being evaluated as partial or total substitutes for traditional ingredients (Freitas, Sousa & Wurlitzer, 2022). In recent years, hemp (*Cannabis sativa* L.) has proven to be a versatile plant with various uses, especially in food products and wellness supplements. Recent research has revealed successful formulations and optimization of kombucha using hempseed as a significant contributor of antioxidants, phenolic compounds, and protein (Reyes-Flores, Pereira & Ramírez-Rodrigues, 2023). Hemp leaves, which are relatively low in cost, offer numerous health benefits. There is a growing interest in hemp leaves because they contain plenty of bioactive compounds, such as flavonoids, phenolics, cannabinoids, alkaloids, and saponins, making them a novel source of foods and drugs, especially for anti-fatigue and antioxidant activities (Zhu et al., 2021).

Additionally, mushrooms have been reported to enhance functionally beneficial health properties, such as inhibiting cytotoxicity, genotoxicity, and improve antioxidant factors (Motafeghi et al., 2023). Milky mushroom (*Calocybe Indica*) is a renowned plant with significant health impacts and medicinal properties, making it a nutritionally valuable edible mushroom. It has been reported to reveal unique activities like fibrinolytic activity, synergism with standard antibiotics, anti-obesity effects, anti-aging, and others (Ghosh & Acharya, 2022). Due to these reasons, both hemp leaves and milky mushrooms can be used as health-improving ingredients in fermented kombucha. The inclusion of SCOBY is important to ensure the complete fermentation process. However, SCOBY, a symbiotic culture between acidophilic yeast, AAB, and LAB, could carry a risk of contamination and may not always be considered entirely safe. Urbahillah, Jayus & Nurhayati (2021) found fungi, namely *Mucor* sp., *Trichoderma* sp., and *Fusarium* sp. in original SCOBY. These fungi, commonly found in soil, plants, and decomposing fruits and vegetables, and some species, are considered opportunistic and benign symbionts of plants. The incorporation of various traditional SCOBYs and diverse ingredients can have distinct effects on the microbial community, ultimately impacting the quality characteristics of kombucha.

Therefore, when using traditional local SCOBY or tea fungus, it is essential to study the microbial consortium to confirm its effect on kombucha characteristics. The objectives of this work were to evaluate whether the microbial communities change during fermentation when using hemp leaves and milky mushrooms flour with local traditional SCOBY. Their effects on kombucha quality, especially phytonutrients and microbial consortium, were evaluated.

## Materials and Methods

### Kombucha ingredients preparation

The hemp leaves (RPF three species) were collected from Burapha University, Chanthaburi Campus, Thailand. The leaves were immediately dried using a tray dryer (Euro Best Technology Co., Ltd., Pathum Thani, Thailand) at 50 °C for 150 min, until a moisture content of 11% and an aw value of 0.35 were achieved. The dried leaves had a total phenolic content of approximately 31.00 ± 0.62 mg GAE/g. The leaves were then packed in transparent plastic bags for further fermentation processes. The milky mushrooms, which were grown at Burapha University, Chanthaburi Campus, were washed and rinsed twice with water and soaked in a solution of Calcium Hypochlorite (Ca(ClO)_2_) (Chlor and Chem International Limited Partnership, Nakhon Sawan, Thailand) with a concentration of 75 ppm for 5 min (Mishra, Abrol & Dubey, 2018). Afterward, the mushrooms were dried using a tray dryer at 60 °C for 18 h, then ground and packed in transparent plastic bags. The ground samples contained important oligosaccharides, such as maltose (36.59/100 g), isomaltose (0.52/100 g), maltotriose (0.16/100 g), isomaltotriose (0.02/100 g), xylose (16.57/100 g), xylobiose (31.91/100 g), xylotriose (17.40/100 g). The obtained substrates were then used for further fermentation. Dried red tea leaves (YOKU company limited, Bangkok, Thailand) and pandan leaves (Baanrakdin, Limited Partnership, Kanchanaburi, Thailand) were obtained from local stores in Thailand that conform to production standards.

### Sectioning of traditional local kombucha SCOBY

Traditional starter culture (SCOBY from a back-slopping method) is used as a microbial consortium for fermenting kombucha products locally in Thailand. A solid-phase SCOBY (approximately 120 cm in diameter) was sourced from a local market in Thailand (Northlandtea Limited Partnership, Lamphun, Thailand). The SCOBY had previously been used to perform 14-days kombucha fermentation and was then stored at 4 °C, following the method of Tran et al. (2020).

### Fermentation of tea broth and sampling

A broth was prepared using red tea leaves 0.75% (w/v), with the following protocol: 78.25% (w/v) of reverse osmosis water was boiled for 5 min, then 10% (w/v) of sucrose (Pacific Sugar Corp., Ltd., Bangkok, Thailand), 0.50% (w/v) of pandan leaves and 0.50% (w/v) of dried hemp leaves were added and steeped for 10 min. After that, all the leaves were removed, and the sweetened tea broth was cooled to room temperature. Then, 10% (w/v) of the local mother SCOBY was inoculated into the tea mixtures and incubated (T1; control treatment) at 30 °C. The recipe with the addition of milky mushroom flour (T2; treatment supplemented with milky mushroom flour) followed the same steps as T1, except the milky flour was added together with granulated sugar. After that, the jar was covered with a sterile tight weave towel, and secured with a rubber band. The mixture was allowed to sit undisturbed with at 30 °C, out of direct sunlight. Samples were taken at initial fermentation (0 h), and after 7, 14 and 21 days under aseptic conditions and used for further analysis.

### Analysis of technological quality

#### pH and total soluble solids

A 100 mL of fermented tea broth was collected from each treatment, and the acidity of the tested samples was determined using a calibrated pH meter (Mettler Toledo FE20, Zürich, Switzerland). The total soluble solids of the samples were measured using refractometry method (Atago handheld refractometer N-1E, Tokyo, Japan). Analyses were performed in triplicate for all samples.

#### Total phenolic content

The total phenolic content was determined using the Folin-Ciocalteu reagent, following the methodology of Zubaidah et al. (2018), with gallic acid utilized as the standard. In a test tube, 1 mL of methanolic extract from the samples was combined with 1.5 mL of the Folin-Ciocalteu reagent (Sigma Aldrich, Burlington, MA, USA) and mixed vigorously using a mixer (Nissin mixer N-20 M) for 15 s. The mixture was then left to stand at room temperature (25 °C) for 5 min before adding 1.5 mL of 0.57 M Sodium Carbonate (Na_2_CO_3_; Sigma-Aldrich, Burlington, MA, USA) and further incubated for 90 min at room temperature (25 °C). Absorbance was measured at 750 nm using a spectrophotometer (UV-1601; Shimadzu, Kyoto, Japan), with methanol replacing the sample extract as the blank in the same mixture. The total phenolic content was quantified and expressed as mg GAE (Gallic Acid Equivalent; Sigma-Aldrich, Burlington, MA, USA) per mL.

#### Total flavonoid content

The total flavonoid content was determined using a colorimetric assay (Nadiah & Uthumporn, 2015). Initially, 100 µL of each sample was combined with 4 mL of distilled water. Subsequently, 0.3 mL of 5% Sodium nitrite (NaNO_2_; Sigma-Aldrich, Burlington, MA, USA) was added followed by 0.3 mL of 10% of Aluminum chloride (AlCl_3_, KemAus, Cherrybrook, Australia) after 5 min. After a further 5 min, 2 mL of 1 M sodium hydroxide (NaOH; KemAus, Cherrybrook, Australia) was added into the mixture. Immediately following that, the mixture was diluted with 3.3 mL of distilled water and thoroughly mixed. Absorbance was then measured at 510 nm using catechin (Sigma-Aldrich, Burlington, MA, USA) as a standard calibration curve. The total flavonoid content of the sample was quantified and expressed as mg catechin equivalents per mL of the sample (mg CE/mL).

#### DPPH analysis

The antioxidant activity was determined using the DPPH radical scavenging activity method, as described and modified by Zubaidah et al. (2018). A 100 µL sample from each treatment of fermented tea was diluted with 3 mL of 96% ethanol, followed by the addition of 1 mL of 0.2 mM DPPH (2,2-Diphenyl-1-picrylhydrazyl; Sigma-Aldrich, Burlington, MA, USA) and thorough homogenization. The reaction mixture was vortexed and allowed to stand in a dark room for 30 min. Subsequently, the absorbance of the sample was measured at 517 nm using a UV-VIS spectrophotometer (UV-1601; Shimadzu, Kyoto, Japan). Control samples were prepared by substituting the sample with distilled water. The antioxidant capacity was calculated as the percentage of DPPH radical scavenging ability using Formula (1)

(1) $${\rm DPPH\, scavenging\, effect\, \%\, Inhibition} = \displaystyle{{\left( {A0 - A1} \right)} \over {A0}} \times 100$$

where A0 is the absorbance of control, and A1 is the absorbance of standard or sample.

#### Enumeration of total viable bacteria

The microbial population changes during tea broth fermentation were monitored by measuring the viable cell count of total bacteria cells. For each sample, a series of 10-fold dilutions (10^1^–10^8^) were prepared using sterile sodium chloride (Himedia, Maharashtra, India) solution (0.85%, w/v) as diluent. A 100 μL aliquot of each sample was spread on PCA (Plate count Agar, Himedia, Maharashtra, India) and then incubated at 35 °C for 24 h. The number of bacteria was expressed as log CFU/mL.

#### Enumeration of lactic acid bacteria (LAB)

Lactic acid bacteria (LAB) was determined using a deep inoculation method with a selective culture medium manufactured by MRS Agar (de Man, Rogosa, and Sharpe Agar; Himedia, Maharashtra, India) as described by Neffe-Skocińska et al. (2017). A series of 10-fold dilutions (10^1^–10^8^) of each sample was prepared using sterile sodium chloride solution (0.85%, w/v) as diluent. A 100 μL aliquot of each sample was spread on MRS agar plates, which were then incubated in anaerobic jars at 35 °C for 48 h. The number of total lactic acid bacteria was expressed as log CFU/mL.

#### Enumeration of fungi

A spread-plate was used to count the total number of yeast and mold. This experiment used PDA (potato dextrose agar, Himedia, Maharashtra, India) as a culture medium, as developed (Wang et al., 2023). The sample was diluted with 9 mL of sterile 0.85% sodium chloride solution to obtain a 10-fold dilution. A 100 μL aliquot of each dilution was spread on PDA plates, which were then incubated at 25 °C for 5 days. The amounts of yeast and mold were counted and calculated as log CFU/mL.

#### Analysis of fungal community by ITS sequencing

Samples were collected from each treatment (T1 and T2) at 0, 14, and 21 days of tea broth fermentation. The samples were kept in 1.5 mL microcentrifuge tubes, and stored at −20 °C. Metagenomic analysis of all samples, with three replicates, was performed to identify fungi.

High quality good yield DNA was extracted from the kombucha samples using PureLink Microbiome DNA Purification Kit (Thermo Fisher Scientific, Waltham, MA, USA), and used as template ITS for metagenomic analysis using the Illumina MiSeq platform (Illumina, San Diego, California, USA). The library was made by amplifying the targets ITS1 (CTTGGTCATTTAGAGGAAGTAA) (Kaashyap, Cohen & Mantri, 2021). The PCR conditions used for ITS1 amplification were: denaturation at 94 °C for 4 min, followed by 35 cycles of denaturation at 94 °C for 30 s, annealing at 50 °C for 1 min, and extension at 72 °C for 1 min and 30 s. Equal concentrations of ITS amplicons were pooled, cleaned with AMPure and assessed using an Agilent 2100 Bioanalyser (Agilent technologies, Santa Clara, CA, USA) to determine purity and to ensure the absence of primer dimers. Sequencing of ITS-1 rDNA ribosomal amplicons was performed using a 454 Genome Sequencer FLX Titanium System (Roche Diagnostics Ltd.) at Teagasc Food Research Centre, Moorepark, according to 454 protocols.

### Statistical analysis

Experiments were conducted in triplicate. Differences in means of technological quality parameters were analyzed using analysis of variance (ANOVA) followed by Duncan’s multiple range test (DMRT), with IBM SPSS statistics 29 (Armonk, New York, USA). A probability level of *P* < 0.05 was considered statistically significant.

### Data availability

Raw Illumina sequence data for all metagenomes have been deposited in the NCBI Sequence Read Archive in BioProject PRJNA1082283.

## Results and Discussion

### Physicochemical quality changes in Kombucha during fermentation

**Figure 1:**
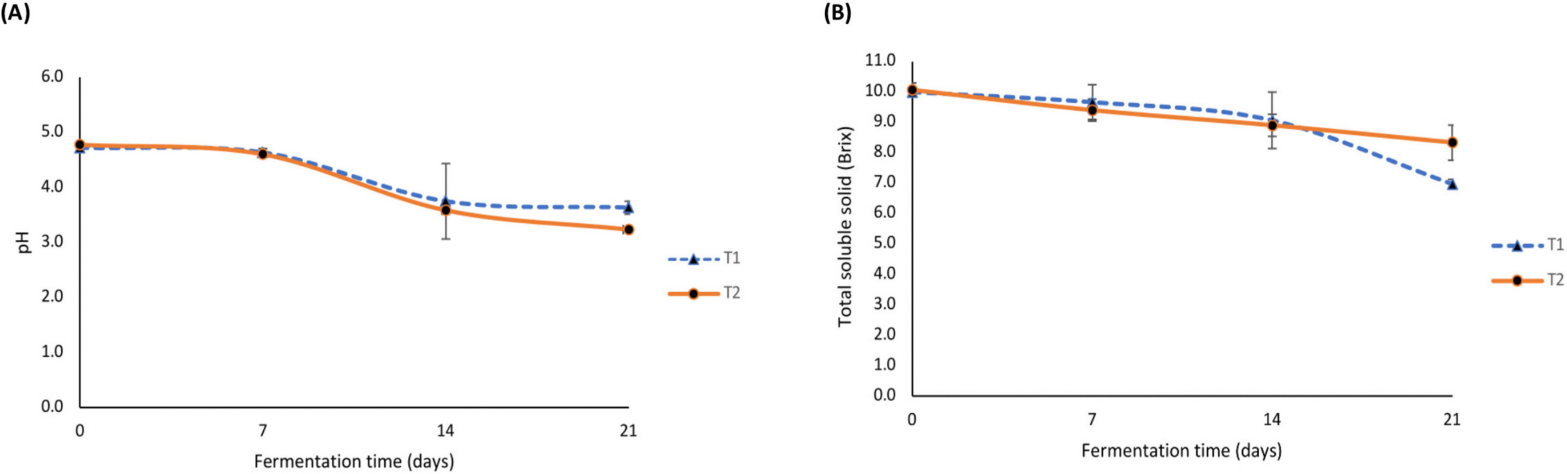
Changes in pH and total soluble solids (Brix) of fermented tea broth samples during fermentation. pH (A), total soluble solids (B). T1 represents the kombucha produced with the addition of hemp leaves (control), and T2 represents the kombucha produced with the addition of hemp leaves and milky mushroom flour. Error bars show the range of the data (*n* = 3).

The measurement of pH determines the acidity or basicity of a solution by indicating the concentration of hydrogen ions (H^+^) in the solution. The changes in pH values of the tested tea broth samples are shown in Fig. 1A. In general, the pH values of all samples decreased with an increase in the fermentation time. Interestingly, both treatments indicated a rapid decrease in pH after 14 days of fermentation; however, the pH value only slightly decreased between 14 and 21 days. T2 samples (kombucha produced with the addition of hemp leaves and milky mushroom flour) fermented for 21 days revealed the lowest pH value of 3.23. Meanwhile, T1 (kombucha produced with the addition of hemp leaves (control treatment)) showed a pH value of approximately 3.63. Similarly, de Noronha et al. (2022) stated that the pH of kombucha stabilizes around 10 days of fermentation due to the buffer effect caused by the formation of organic acids and carbon dioxide. Normally, pH is a key factor that controls the proper course of fermentation and is used to determine the end of the process (Neffe-Skocińska et al., 2017). Moreover, Vohra et al. (2019) suggested that a pH range of 2.9–4.5 is vital for the activity of AAB and yeast, which play an important role in fermentation and the properties of the beverage. Similarly, Antolak & Kręgiel (2015) stated that a pH below 5.4 facilitates yeast survival and optimal growth; however, this can vary depending on the species and strain, with an average range of 4.5–6.5. Also, the acidity of kombucha, mainly generated from organic acids through microbial metabolism, is usually affected by the carbon source, nitrogen source, and microbial community (Zou et al., 2021).

Total soluble solids (TSS) commonly represent the organic acids and sugar content in a solution. The higher the total acid formed, the lower the pH value; as the acid in the kombucha releases protons (H^+^), decreasing the pH value. Both total sugar and soluble solids decrease during the fermentation process (Jayabalan et al., 2014). As shown in Fig. 1B, T1 showed a decrease in TSS from the initial phase to 14 days of fermentation, followed by a rapid decrease to 7 Brix by day 21. On the contrary, T2 showed a slight decrease in TSS during fermentation, starting at 10.10 Brix and reducing to 8.30 Brix by day 21. Similarly, Zubaidah, Ifadah & Afgani (2019) reported that kombucha produced with supplementation of fruits exhibited a slower decrease in TTS. Additionally, microorganisms use sucrose as the major substrate to produce various organic acids during fermentation, resulting in the decrease of total soluble solids in kombucha (Kaewkod, Bovonsombut & Tragoolpua, 2019).

### Phytonutrient quality changes in kombucha during fermentation

Phytonutrient and antioxidant assays were performed in the present study to assess the improvement of these metabolites during fermentation with hemp leaves and milky mushroom flour as ingredients, and to evaluate their effect on microbial population. Tea broth samples that were prepared for immediate use, as well as and kombucha that had fermented for 0, 7, 14, and 21 days were used for all analyses. According to the data presented in Table 1, the total phenolic content of kombucha showed a significant difference (*p* < 0.05) throughout 0–21 days of fermentation for both T1 and T2. The total phenolic content (TPC) in both fermentation treatments gradually increased over time, with the kombucha supplemented with milky mushroom achieving the highest TPC at 155.91 mg GAE/mL (21 days).

**Table 1:** Changes in phytonutrients and DPPH radical scavenging activities of kombucha during fermentation.

Fermentation times (days)	Total phenolic content (mg GAE/mL)		Total flavonoid content (mg CE/mL)		DPPH radical scavenging activities (percentage inhibition, %)	
	T1	T2	T1	T2	T1	T2
0	92.02^d^ ± 0.16	124.80^c^ ± 0.16	0.63^c^ ± 0.01	0.77^c^ ± 0.05	89.59^c^ ± 0.12	90.26^b^ ± 0.22
7	119.71^c^ ± 0.00	125.08^c^ ± 1.63	0.86^a^ ± 0.01	0.93^b^ ± 0.02	89.91^bc^ ± 0.12	84.21^d^ ± 0.34
14	124.62^b^ ± 0.32	141.84^b^ ± 11.30	0.68^b^ ± 0.03	0.96^ab^ ± 0.02	90.26^b^ ± 0.16	84.98^c^ ± 0.44
21	129.52^a^ ± 0.43	155.91^a^ ± 0.16	0.69^b^ ± 0.03	1.01^a^ ± 0.04	91.95^a^ ± 0.64	91.03^a^ ± 0.28

**Note:**

T1 = Red tea leaves, pandan leaves, sucrose, and hemp leaves (control treatment), T2 = Red tea leaves, pandan leaves, sucrose, hemp leaves, and milky mushroom flour. Values with different superscripts in a column represent significant differences (*p* < 0.05).

The total flavonoid content (TFC) in both treatments showed a pattern of increasing differences. T1 exhibited a concentration of 0.86 mg CE/mL at 7 days, with a slight decrease thereafter, indicating a significant difference between the fermentation periods (*p* < 0.05). On the other hand, T2 exhibited an increase in the concentration over time, reaching the highest concentration of 1.01 mg CE/mL at 21 days. Therefore, phytonutrients such as TPC and TFC in kombucha produced with hemp leaves supplemented with milky mushroom flour were mainly released after 7 days of fermentation in the kombucha liquid phase.

The antioxidant activity expressed in DPPH radical scavenging activities (percentage inhibition, %) is presented in Table 1. The data showed an increase in the antioxidant activity as the fermentation process progressed. The antioxidant activity profile had a similar evolution pattern to that of the TPC. The percentage inhibition of DPPH free radical scavenging activity increased continuously from the start of fermentation to 21 days. Particularly, at the end of the fermentation period, T1 and T2 showed the highest percentage inhibition at 91.95% and 91.03%, respectively. Similarly, studies by Chakravorty et al. (2016) and Jakubczyk et al. (2020), and Zhou et al. (2022) suggested an increase in the total phenolic content during kombucha fermentation process. Notably, tea fungi play an important role in the fermentation process, where microbial impacts the conversion of chemical components to phenolic metabolites (Zhu et al., 2020; Utoiu et al., 2018). Moreover, Reyes-Flores, Pereira & Ramírez-Rodrigues (2023) reported that kombucha produced from hemp can have enhanced phenolic content, with these compounds progressively increasing with an increase in the amount of hempseed. Phenolic compounds are well-known for their high levels of antioxidants because of their ability to scavenge free radicals of oxygen, superoxide free radicals, and hydroxyl radicals. Thus, the increased concentration of phenolic compounds in kombucha samples may indicate improved antioxidant capacity (Ahmed et al., 2019; Jayabalan et al., 2008). In addition, milky mushrooms have been reported to possess high levels of phenolics and exhibit a significant capacity to inhibit radical scavenging activity on DPPH (Mishra, Pal & Arunkumar, 2014). Regarding fermentation time, our research corresponds to Pure & Pure (2016), who reported that fermentation time influenced the factors associated with a marked increase in polyphenol content and antioxidant activity over a period of 20 days. Moreover, Ivanišová et al. (2019) detected flavonoids in kombucha, at a concentration of approximately 0.13 mg QE/mL. Similarly, de Noronha et al. (2022) suggested that TFC could decrease during prolonged fermentation. Their findings showed that the initial value of TFC in kombucha was 2.3 ± 0.2 QE mg/mL, which then reduced by 50% (1.1 ± 0.3 µg QE/mL) and remained constant throughout the experimental period. Chen & Sang (2014) reported that flavonoid compounds may undergo degradation by enzymes, such as β-glucosidase, which are released by microorganisms, leading to the formation of smaller molecules. Hsieh, Chiu & Chou (2021) reported that fermentation of black tea contributed to the degradation of flavonoids. For these reasons, hemp leaves and milky mushrooms can be used as functional ingredients to enhance phytonutrients composition in kombucha. Meanwhile, a longer fermentation period leads to greater stability and retention of flavonoids.

### Dynamics of microorganisms during Kombucha fermentation

**Figure 2:**
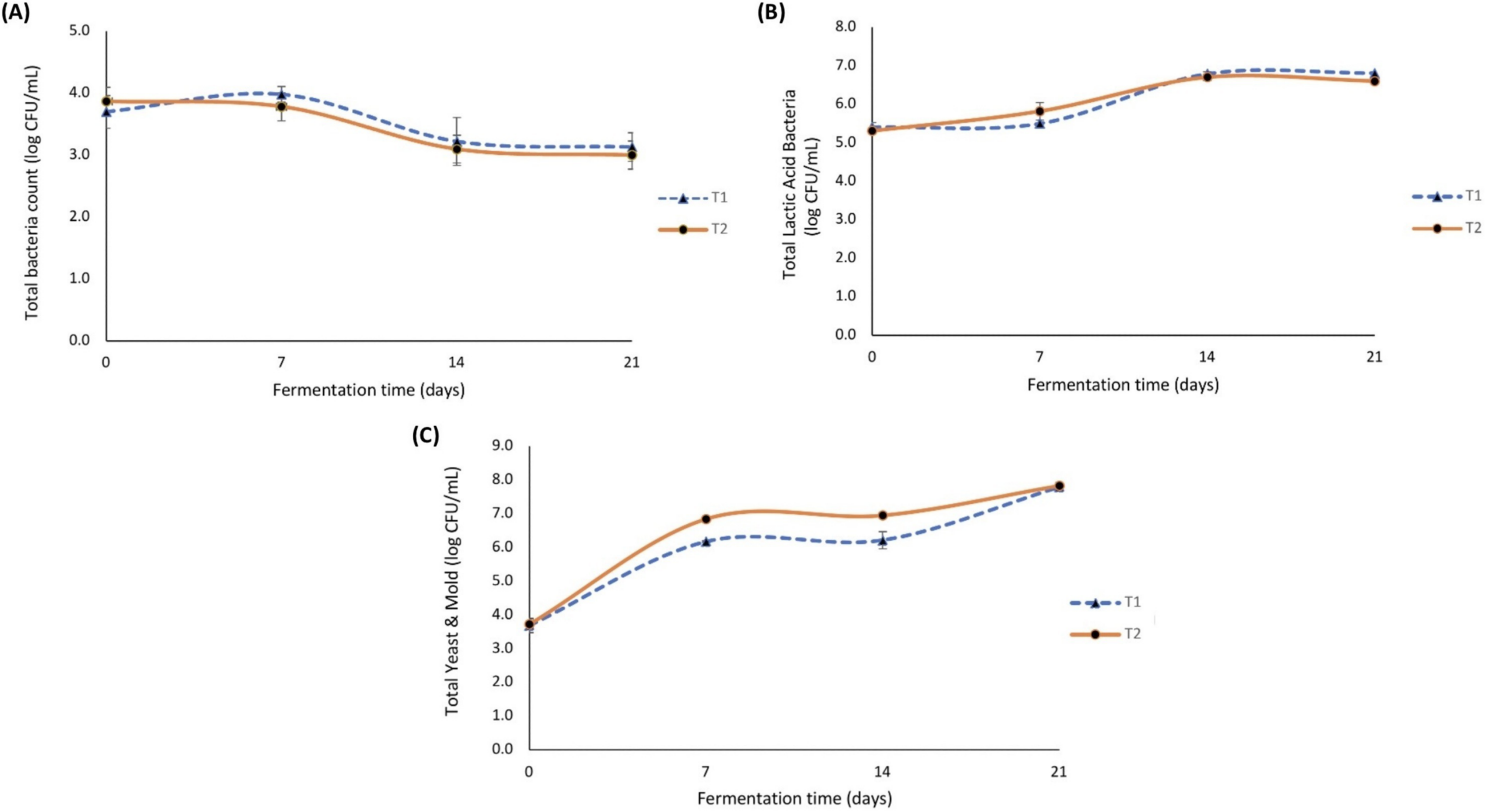
Changes in microbial populations during tea broth fermentation. Total bacteria count (A), total lactic acid bacteria count (B), and total yeast and molds count (C). T1 represents the kombucha produced with the addition of hemp leaves (control), and T2 represents the kombucha produced with the addition of hemp leaves and milky mushroom flour. Error bars show the range of the data (*n* = 3).

Kombucha is a fermented beverage produced using acid-tolerant species of a symbiotic culture of bacteria and yeasts. Traditionally, tea leaves and sugar are the main substrates for kombucha fermentation. However, this research evaluated modifications by adding hemp leaves and milky mushroom flour as substrates alongside red tea leaves. In addition, the genera of microorganisms and the safety of traditional local SCOBY used in this study have not yet been confirmed. Therefore, it is necessary to monitor the changes in microorganisms during fermentation, as these can affect the quality and safety of kombucha. The total numbers of bacteria, lactic acid bacteria, and yeast & mold over 21 days of fermentation are shown in Figs. 2A–2C, respectively. The total bacteria count of both tea broths remained relatively unchanged during the first 7 days, ranging from 3.70 to 3.98 log CFU/mL. By the 14^th^ day, both T1 and T2 showed a significant decrease in the number of bacteria, reducing to approximately 3.22 and 3.10 log CFU/mL, respectively. By the end of the fermentation, the total bacteria count remained steady within the range of 3.00–3.13 log CFU/mL. On the contrary, the total lactic acid bacteria count for all treatments increased steadily during the first 14 days of fermentation. T1 and T2 showed rapid increases to 6.79 and 6.70 log CFU/mL on the 14^th^ day, respectively, with a slight decrease thereafter. Interestingly, changes in total yeast and mold during fermentation for all treatments rapidly increased during the first 7 days, from 3.69–6.84 log CFU/mL. After 7 days, the counts remained stable until the 14^th^ day. Subsequently, the total yeast count slightly increased to a range of 6.21–7.82 log CFU/mL by the end of fermentation, with T1 reaching 7.78 log CFU/mL and T2 reaching 7.82 log CFU/mL. The trend of total bacteria count obtained in this research was found to be similar to that of Klawpiyapamornkun et al. (2023), who found that adding gooseberry as a substrate resulted in a slight increase in total bacteria during the first 3 days, followed by a decrease by the end of fermentation. Zhao et al. (2018) reported that the total count of bacteria initially increased rapidly by the 5^th^ day, then gradually decreased by the 14^th^ day. When comparing the trends of total bacteria count and yeast and mold count, contrasting results were observed. Our research showed that yeast and mold count rapidly increased during the initial fermentation and remained slightly high until the end of fermentation. Similarly, Matei et al. (2018) indicated that during the first 6 days of fermentation, the level of yeast rapidly increased, with concentrations remaining practically constant from the 10^th^ day of the fermentation to the end of the process (18 days). Zhao et al. (2018) found that total count of yeasts peaked at 7 days, thereafter remaining stable until the end of fermentation. These trends are supported by Tapias et al. (2022), who described the cooperative, competitive, but overall symbiotic relationship of yeast and bacteria in kombucha. These correlate with the initial 3 days of fermentation, where cellulose yield increases due to favorable fermentation conditions and the synergistic microbial metabolism of yeast and AAB. Thereafter, yeast breaks down sucrose into glucose and fructose using invertase enzymes. These products can be used by yeast to produce ethanol, as well as by AAB for cellulose biosynthesis and organic acid synthesis (Leonarski et al., 2021; Tapias et al., 2022; Ayed, Ben Abid & Hamdi, 2017). Thus, it can be seen that the pH values of all kombucha beverages gradually decrease, and the accumulation of ethanol and organic acids produced by yeast inhibits the growth of other bacteria. Moreover, Marsh et al. (2014) suggests that the microbial diversity within the kombucha consortium is influenced by various factors, which include the fermentation times and the geographic origin of the SCOBY, which is consistent with our results. As for the total lactic acid bacteria, the population of LAB increased with an increase in the fermentation time, similar to the trend observed for yeasts. Likewise, Coton et al. (2017) reported that during fermentation, LAB could survive under acidic, aerobic, and static conditions, embedded in a floating biofilm for an average of 8 to 15 days. Neffe-Skocińska et al. (2017) reported a similar observation, with LAB counts increasing during fermentation and peaking after 10 days.

### Fungal community during Kombucha fermentation

**Figure 3:**
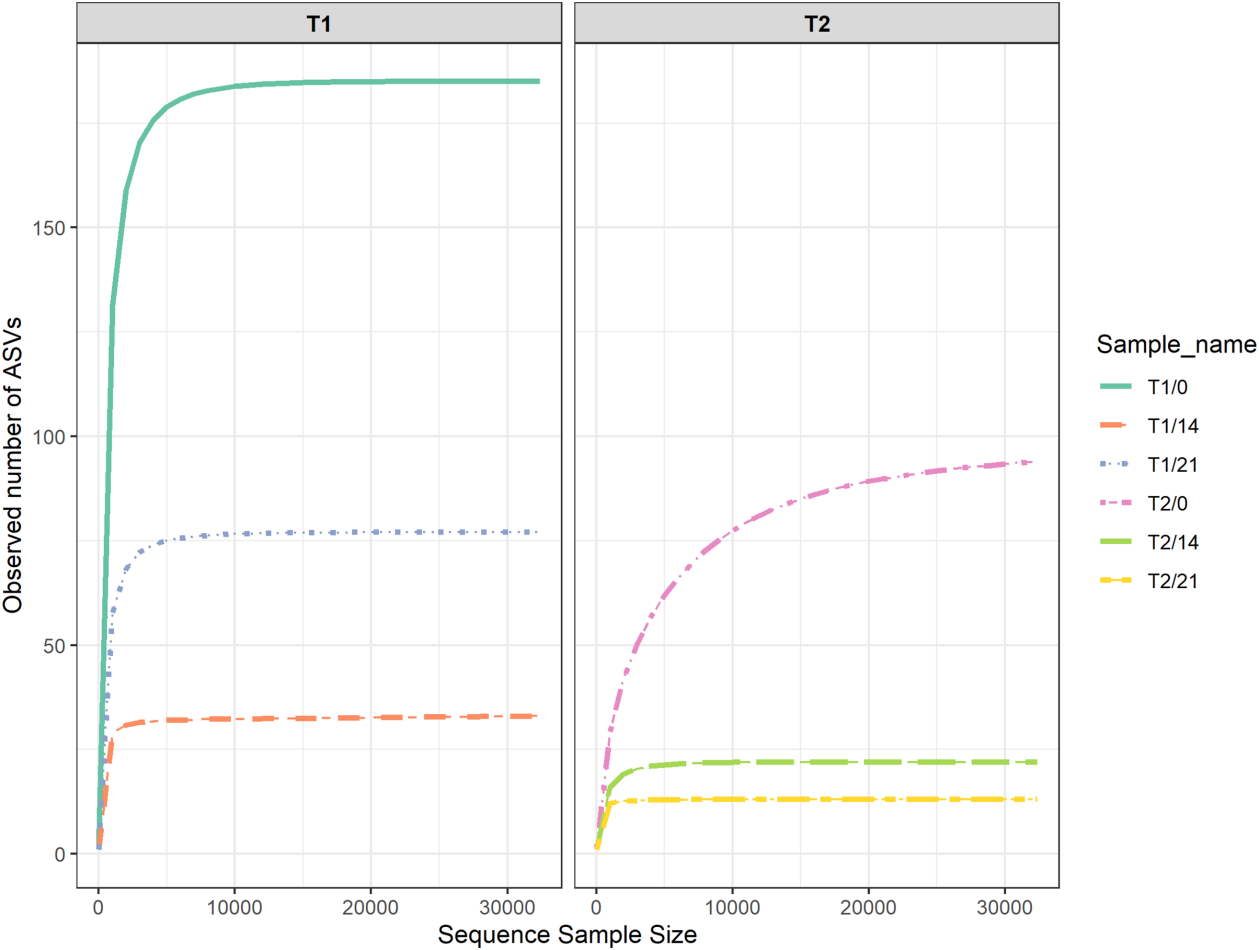
Rarefaction curves of kombucha during 21 days of fermentation, comparing T1 (kombucha produced with the addition of hemp leaves, control) and T2 (kombucha produced with the addition of hemp leaves and milky mushroom flour). Green curves correspond to T1 at 0 days, orange curves correspond to T1 at 14 days, blue curves correspond to T1 at 21 days, pink curves correspond to T2 fermented at 0 days, lark green curves correspond to T2 at 14 days, and yellow curves correspond to T2 at 21 days.

**Figure 4:**
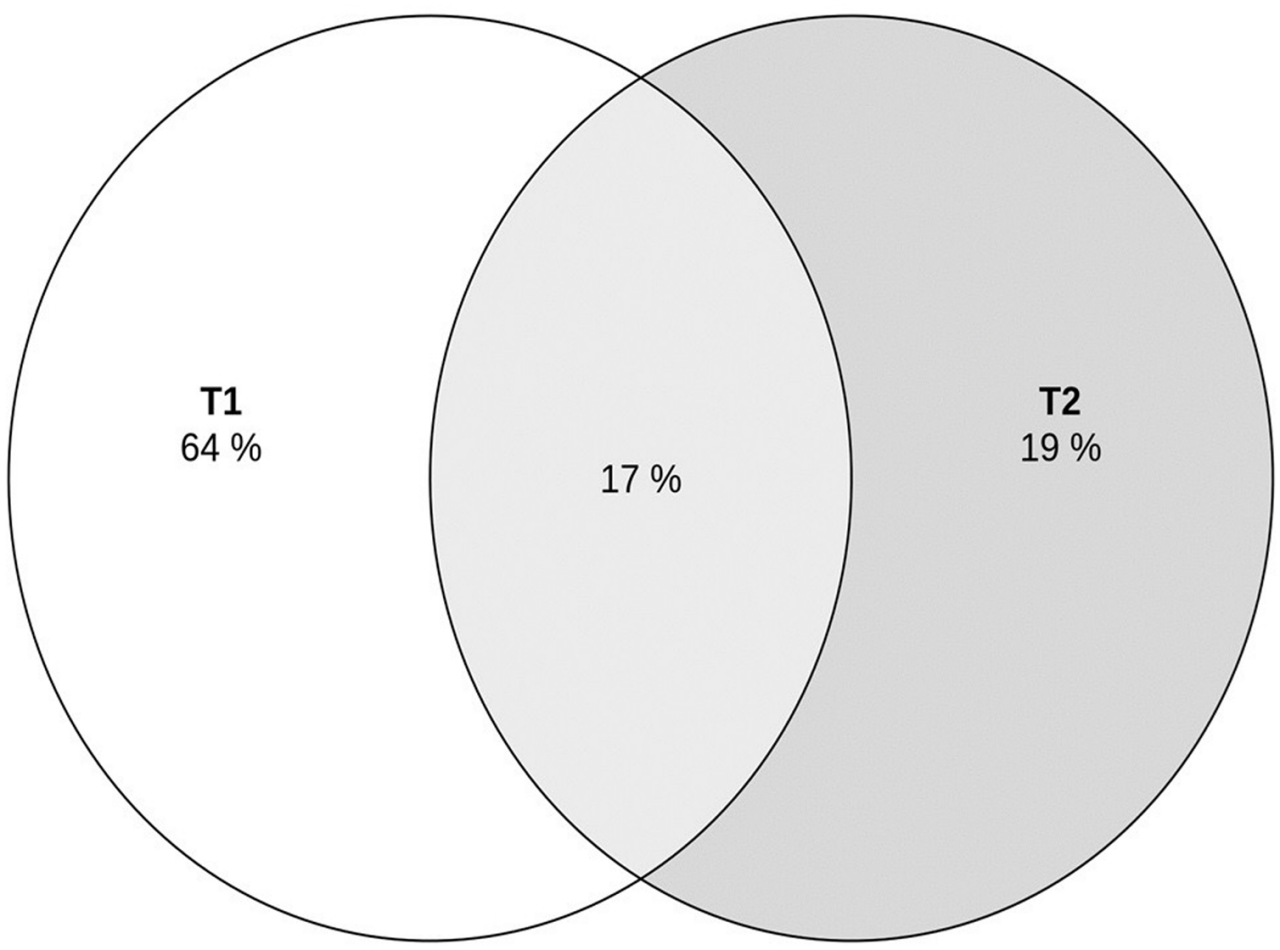
Venn diagram illustrating unique and shared genera of microbial communities during kombucha fermentation under different ingredient treatments. T1 (kombucha produced with the addition of hemp leaves (control)) and T2 (kombucha produced with the addition of hemp leaves and milky mushroom flour).

Rarefaction curves of each sample (Fig. 3) demonstrate the relationships between the number of amplicon sequencing variants (ASVs) and amplicons in the samples. The relationship appeared to be linear, with small fractions of the number of ASVs revealed, and an asymptotic trend observed when most ASVs were present in the samples. This might indicate that the samples adequately represented the microbial diversities (de Jong et al., 2022). Venn diagrams illustrated in Fig. 4 show that 64% and 19% of the genera were unique to T1 and T2, respectively, while 17% of the total genera were shared between T1 and T2. The experimental results indicate that the diversity of microorganisms in the sample of fermented tea with added milky mushroom flour was lower than that of the control sample. This suggests that the initial substance may have limited the growth of fungi and AAB. During kombucha fermentation process, the SCOBY consumes not only the sugar in the tea but also other ingredients. Based on this process, it could be suggested that soluble ingredients should be considered. This corresponds to Thejaswini et al. (2013), who explained that using milky mushroom extracted using strong alkali revealed constituent sugars such as rhamnose, arabinose, xylose, galactose, and glucose, while hot water extract had lower sugar concentrations. Oligosaccharides are less soluble in aqueous alcohol solutions compared to monosaccharides, and their solubility decreases as the number of monosaccharide units increases. Similar results were reported by Zartl et al. (2018), who found that raffinose family oligosaccharides (RFOs) were not fermented, and some RFOs such as trisaccharide raffinose and tetrasaccharide stachyose delayed and limited fermentation.

**Figure 5:**
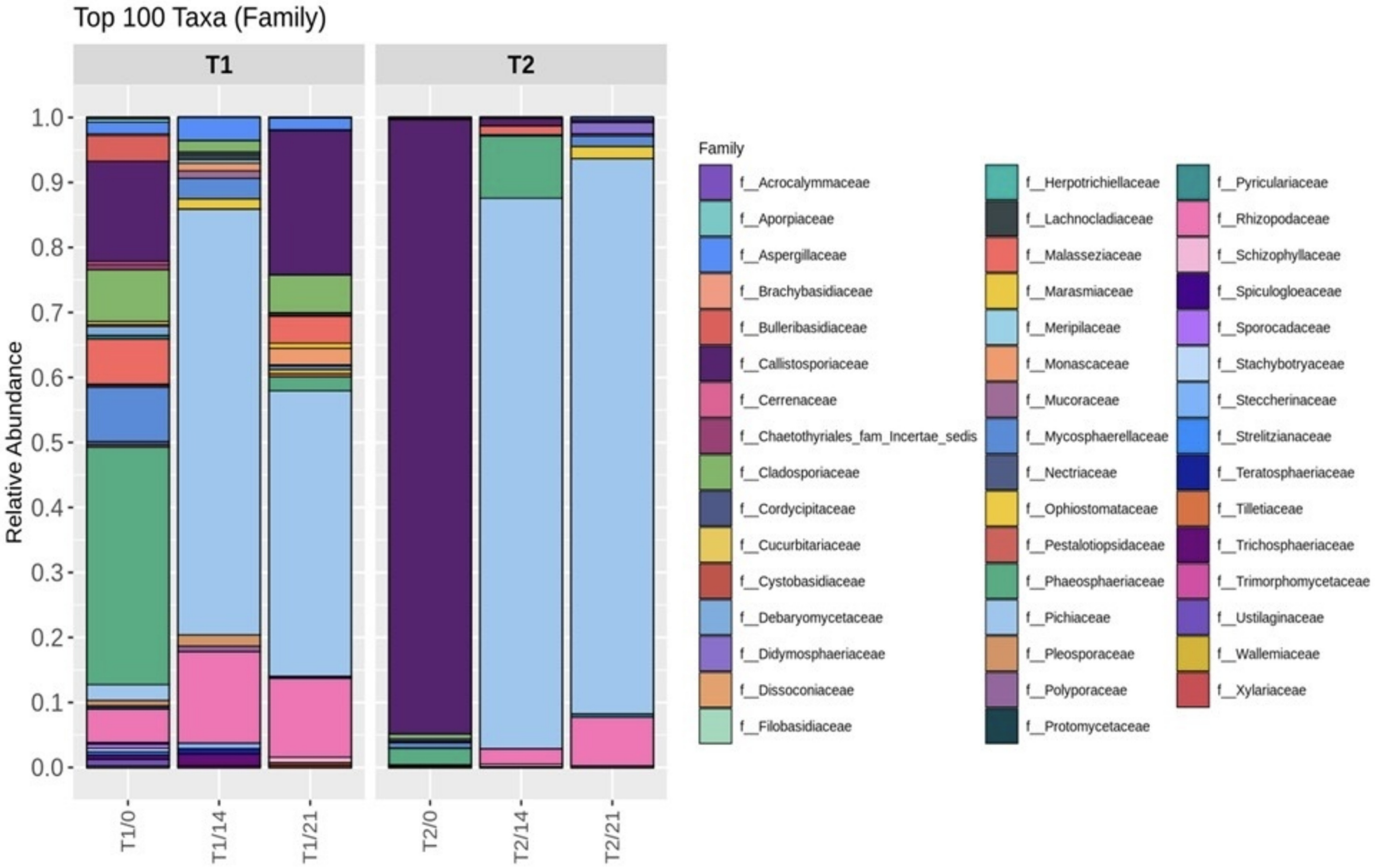
Relative abundance of microorganisms at the family level during kombucha fermentation under different ingredient treatments. T1 (kombucha produced with the addition of hemp leaves (control)) and T2 (kombucha produced with the addition of hemp leaves and milky mushroom flour) on days 0, 14, and 21 of fermentation.

The relative abundance of family is depicted in Fig. 5. One hundred taxa at the family level obtained in the experiment were identified. On day 0, the top eight families in T1 were *F_ Phaeosphaeriaceae* (33.13%), *F_Callistosporiaceae* (13.82%), *F_Mycosphaerellaceae* (8.42%), *F_ Cladosporiaceae* (7.35%), *F_ Malasseziaceae* (6.22%), *F_ Bulleribasidiaceae* (4.69%), and *F_ Rhizopodaceae* (4.56%). Meanwhile, the predominant families in T2 were: *F_Callistosporiaceae* (93.79%), *F_Phaeosphaeriaceae* (2.51%), *F_ Mycosphaerellaceae* (0.88%), *F_ Cladosporiaceae* (0.76%), and *F_ Cordycipitaceae* (0.38%). However, *F_Pichiaceae* (>0.42) was predominant in both T1 and T2 at 21 days of fermentation. From this study, we can infer that the dominant yeast in this community is similar to those described previously by Kahraman-Ilıkkan (2023), where *Pichia kudriavzevii* (64.35%) was identified as the dominant yeast in kombucha after 7 days of fermentation in both the liquid tea and pellicle. Similarly, Kilmanoglu, Cinar & Durak (2024) revealed that in monocultures and cocultures during fermentation, *Pichia* and *Brettanomyces* were dominant, reaching their highest values on day 7 of fermentation. Moreover, it has been reported that *Pichia kudriavzevii* is the predominant yeast in kombucha, with interesting metabolic patterns for sugars such as D-Glucose, L-sorbose, and D-Xylose (Wang et al., 2022). These metabolic patterns are well-adapted to describe the carbohydrate profile in hemp leaves, consisting of glucan and xylan between 32.63 to 44.52% and 10.62 to 15.48% respectively, along with monomeric sugars glucose and xylose (Viswanathan et al., 2020).

**Figure 6:**
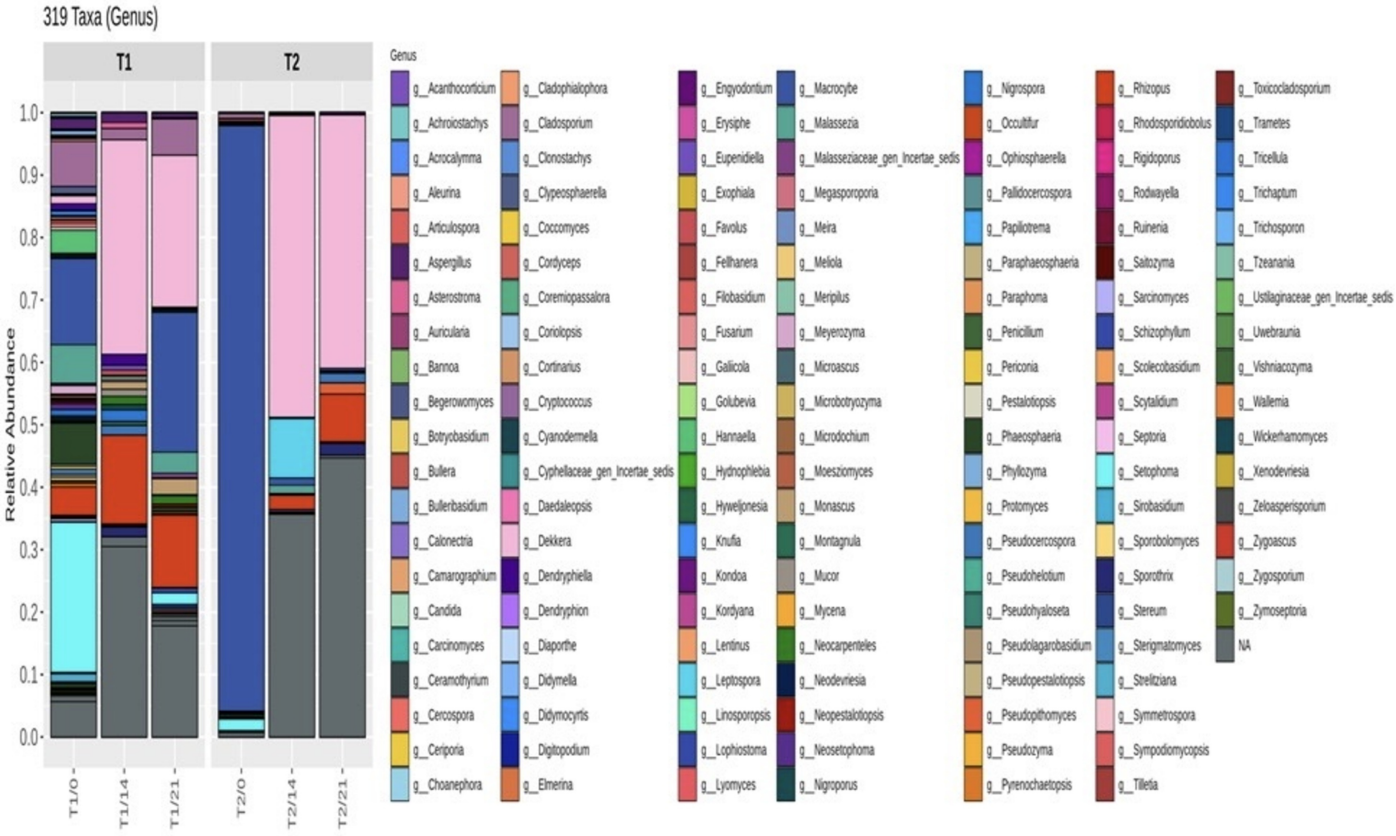
Relative abundance of microorganisms at the genus level during kombucha fermentation under different ingredient treatments. T1 (kombucha produced with the addition of hemp leaves (control)) and T2 (kombucha produced with the addition of hemp leaves and milky mushroom flour) on days 0, 14, and 21 of fermentation.

Figure 6 displays the relative abundance of different genera in each treatment. Three hundred and nineteen at the genera level were specifically identified. On day 0, the top five genera in T1 were *g__Setophoma* (25.91%), *g__Macrocybe* (14.88%), *g__Cladosporium* (7.81%), *g__Phaeosphaeria* (7.12%), *g__Malassezia* (6.63%). Meanwhile, *g__Macrocybe* (94.55%), *g__Setophoma* (1.87%), *g__Cladosporium* (0.77%), *g__Phaeosphaeria* (0.40%), *g__Cordyceps* (0.38%) were the top five genera in T2. On day 21, g__Dekkera had the highest relative abundance in all samples. This finding was in accordance with the results of Marsh et al. (2014), who found that *Dekkera* was the predominant fungus (>30%) during multiple kombucha fermentations. T1 control treatment had relative abundances of *g_Cladosporium*, exceeding 0.7%. Cheng et al. (2024) recently identified *Cladosporium*, with a relative abundance exceeding 1% during kombucha fermentation treated with *Lithocarpus litseifolius*. In addition, the genera *g__Cladosporium*, *g__Monascus*, *g__Setophoma*, *g__Neocarpenteles*, *g__Malasseziaceae*_gen_Incertae_sedis, *g__Phaeosphaeria* were not found in T2 on day 21 of fermentation. Consequently, the growth and survival of the yeast during the fermentation process using hemp leaves and milky mushroom flour indicate that important fungi are found in abundance over the extended fermentation period of 21 days. This is likely due to the chemical components in hemp leaves and mushrooms that affect fungal growth. This results in a variety of fungi that are less abundant compared to other raw materials, especially mushroom flour. Additionally, this might suggest that *Cannabis sativa* L. has an antifungal activity. Cannabis varieties contain the chemotypes I and II, including cannabidiol (CBD) and tetrahydrocannabinol (THC) levels. They could inhibit bacterial cell growth and spore germination of pathogenic fungi (Berardo et al., 2024).

## Conclusions

The present study investigated the use of hemp leaves and milky mushroom flour as alternative functional ingredients in kombucha tea production, utilizing a traditional local SCOBY. The research also examined the dynamic changes in microorganisms during the fermentation process with these raw materials to determine their impact on the quality of the final products. The result showed that both hemp leaves and milky mushroom flour can be effectively used in kombucha production. The total phenolic content and total flavonoid content increased over the fermentation period, with the highest values recorded at day 21. Specifically, kombucha supplemented with milky mushroom flour showed the highest total phenolic content of 155.91 mg GAE/mL and significant antioxidant activity, with a DPPH radical scavenging activity of 91.03%. These findings suggest that the incorporation of hemp leaves and milky mushroom flour not only enhances the phytonutrient content but also improves the antioxidant properties of kombucha, making it a valuable functional beverage with potential health benefits. Additionally, an interesting point developed from the fungal community analysis. It was revealed that the addition of hemp leaves and milky mushroom flour (unique genera 19%) to kombucha fermentation limited the diversity of fungal species compared to hemp leaves without flour (unique genera 64%). The dominant presence of specific fungal *F_Pichiaceae* (>0.42) was observed on fermentation day 21, similar to *g__Dekkera*, which had the highest relative abundance in both treatments. Therefore, the relationship between ingredients and microorganism populations can clarify the fungal population dynamics during fermentation. While the addition of certain ingredients may limit fungal growth, it does not directly affect the release of phytonutrient quality in the kombucha. Addressing the areas outlined above in future research could provide comprehensive insights and practical solutions to optimize the use of these ingredients, ultimately contributing to the development of innovative and health-promoting kombucha products.

## Supplemental Information

10.7717/peerj.18116/supp-1 Supplemental Information 1 Raw data kombucha production.

10.7717/peerj.18116/supp-2 Supplemental Information 2 Raw data ITS.

## Additional Information and Declarations

### DNA Deposition

The following information was supplied regarding the deposition of DNA sequences: The raw Illumina sequence data for all metagenomes are available at the NCBI Sequence Read Archive: PRJNA1082283.

### Data Availability

The following information was supplied regarding data availability: The raw measurements are available in the Supplemental Files.
